# Supplementary material for: Drug repurposing for aging research using model organisms
Source: Aging Cell. 2017 Jun 16;16(5):1006–15. doi: 10.1111/acel.12626 (PMC5595691; doi:10.1111/acel.12626)
Supplement: Supplementary file 7 — Data S1 Zip‐Archive of all report cards. [file ACEL-16-1006-s007.zip › RC_OLA.pdf]

OLA

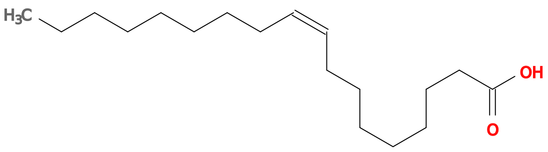

Database identifiers

ChEMBLCompound	CHEMBL8659
CHEBI	16196
eMolecules	476169

Ranking

	Rank	Score
Drosophila	507/697	0.262
C. elegans	NA	NA

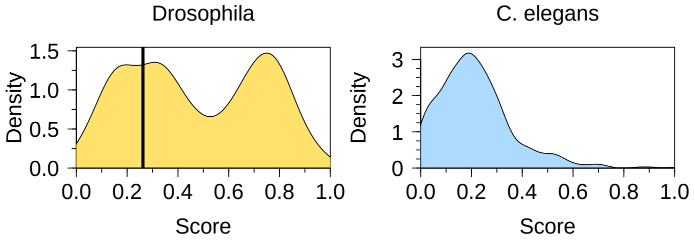

	Ageing implication	Domain conservation	Binding site conservation	Binding affinity	Bioavailability	Lipinski	Promiscuity	Purchasability	Drug approval	**Total**
Drosophila	0.624	0.649	0.664	0.88	(0.9)	-0.05	-0.0	0.1	0.0	0.262
C. elegans	NA	NA	NA	NA	NA	NA	NA	NA	NA	NA

Names

- **oleic acid**
- (9Z)-Octadecenoic acid
- 18:1Delta9cis
- C18:1 n-9
- cis-9-octadecenoic acid
- cis-Delta(9)-octadecenoic acid
- Octadec-9-enoic acid
- Oelsaeure
- Oleate

Roles

Escherichia coli metabolite, plant metabolite, EC 3.1.1.1 (carboxylesterase) inhibitor, solvent, antioxidant, Daphnia galeata metabolite

Status

Approved drug (according to ChEMBL)	No
Number of Rule of 5 violations	1
Binding affinity to original target in log units (RF-Score prediction)	6.99
Burns *C. elegans* bioavailability prediction	-2.89

Compound Target Characteristics

Retinoic acid receptor RXR-alpha

Best gene implication in ageing for this target family came from gene Q0VJ96 via mapping the annotation from RGD 3610 annotated in RGD 2014-03-11. Annotation GO 7568 (aging) was Inferred from Expression Pattern

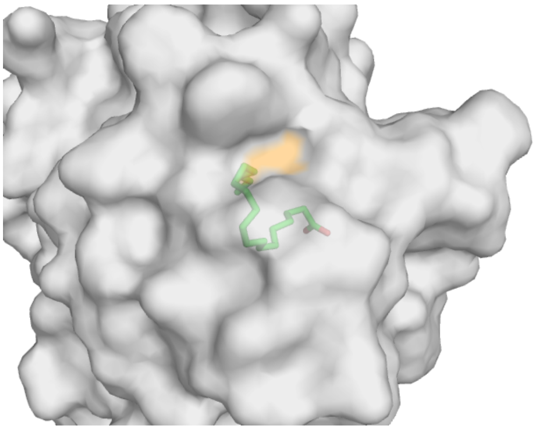

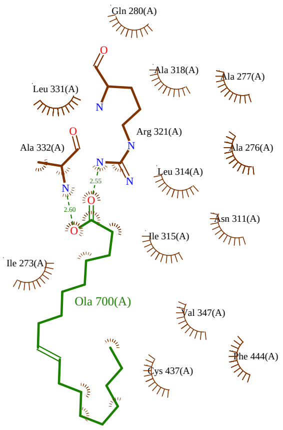

protein	amino acids contacts (binding site)													
PDB:1dkf:chainA:P28700	I	A	A	Q	N	L	I	A	R	L	A	V	C	F
tr:B3KY83:B3KY83_HUMAN	I	A	A	Q	N	L	I	F	R	L	A	V	C	F
sp:P19793:RXRA_HUMAN	I	A	A	Q	N	L	I	F	R	L	A	V	C	F
tr:F1D8Q5:F1D8Q5_HUMAN	I	A	A	Q	N	L	I	F	R	L	A	V	C	F
tr:Q6P3U7:Q6P3U7_HUMAN	I	A	A	Q	N	L	I	F	R	L	A	V	C	F
tr:Q0VJ96:Q0VJ96_RAT	I	A	A	Q	N	L	I	F	R	L	A	V	C	F
tr:Q6LC96:Q6LC96_MOUSE	I	A	A	Q	N	L	I	F	R	L	A	V	C	F
sp:P28700:RXRA_MOUSE	I	A	A	Q	N	L	I	F	R	L	A	V	C	F
tr:A2AJP1:A2AJP1_MOUSE	I	A	A	Q	N	L	I	F	R	L	A	V	C	F
sp:P20153:USP_DROME	L	V	V	Q	I	L	I	F	R	L	N	V	C	F

	whole protein		domain-based		contact-based	
protein	ident	simil	ident	simil	ident	simil
PDB:1dkf:chainA:P28700	1.0	1.0	1.0	1.0	1.0	1.0
tr:B3KY83:B3KY83_HUMAN	0.78	0.78	1.0	1.0	0.93	0.92
sp:P19793:RXRA_HUMAN	0.97	0.99	1.0	1.0	0.93	0.92
tr:F1D8Q5:F1D8Q5_HUMAN	0.97	0.99	1.0	1.0	0.93	0.92
tr:Q6P3U7:Q6P3U7_HUMAN	0.87	0.88	1.0	1.0	0.93	0.92
tr:Q0VJ96:Q0VJ96_RAT	0.99	1.0	1.0	1.0	0.93	0.92
tr:Q6LC96:Q6LC96_MOUSE	0.94	0.94	1.0	1.0	0.93	0.92
sp:P28700:RXRA_MOUSE	1.0	1.0	1.0	1.0	0.93	0.92
tr:A2AJP1:A2AJP1_MOUSE	1.0	1.0	1.0	1.0	0.93	0.92
sp:P20153:USP_DROME	0.37	0.65	0.4	0.66	0.57	0.66

usp (FBgn0003964) associated phenotypes

circadian rhythm defective, germline clone, heat sensitive, hypoactive, lethal - all die before end of pupal stage, lethal - all die during P-stage, lethal - all die during first instar larval stage, maternal effect, neuroanatomy defective, partially, rescuable maternal effect, somatic clone, some die during pupal stage, touch sensitivity defective

(Information from FlyBase)

usp (UniProt:P20153) annotation

Function: Receptor for ecdysone. May be an important modulator of insect metamorphosis. Plays an important part in embryonic and post-embryonic development. Binds to ecdysone response elements (ECRES) such as in the promoter region of s15 chorion gene.

Subunit: Heterodimer of USP and ECR. Only the heterodimer is capable of high-affinity binding to ecdysone. (PubMed:8247157).
Subcellular location: Nucleus.
Domain: Composed of three domains: a modulating N-terminal domain, a DNA-binding domain and a C-terminal ligand-binding domain.

(Information from UniProt)
